# Network-aided Bi-Clustering for discovering cancer subtypes

**DOI:** 10.1038/s41598-017-01064-0

**Published:** 2017-04-21

**Authors:** Guoxian Yu, Xianxue Yu, Jun Wang

**Affiliations:** College of Computer and Information Science, Southwest University, Chongqing, China

## Abstract

Bi-clustering is a widely used data mining technique for analyzing gene expression data. It simultaneously groups genes and samples of an input gene expression data matrix to discover bi-clusters that relevant samples exhibit similar gene expression profiles over a subset of genes. The discovered bi-clusters bring insights for categorization of cancer subtypes, gene treatments and others. Most existing bi-clustering approaches can only enumerate bi-clusters with constant values. Gene interaction networks can help to understand the pattern of cancer subtypes, but they are rarely integrated with gene expression data for exploring cancer subtypes. In this paper, we propose a novel method called Network-aided Bi-Clustering (NetBC). NetBC assigns weights to genes based on the structure of gene interaction network, and it iteratively optimizes sum-squared residue to obtain the row and column indicative matrices of bi-clusters by matrix factorization. NetBC can not only efficiently discover bi-clusters with constant values, but also bi-clusters with coherent trends. Empirical study on large-scale cancer gene expression datasets demonstrates that NetBC can more accurately discover cancer subtypes than other related algorithms.

## Introduction

Gene expression means that cells transfer the genetic information in deoxyribonucleic acid (DNA) into a protein molecule with biological activity through transcription and translation in life process^1^. Microarry techniques enable researchers simultaneously measure expression levels of numerous genes^2^. The measurements of gene expression under many specific conditions are often represented as a gene expression data matrix, of which each row corresponds to a gene and each column represents the expression levels under a specific condition^3, 4^.The specific conditions usually relate to environments, patients, tissues, time points, and they are also synonymously called as samples. One key step of analyzing gene expression data is to identify clusters of genes, or of conditions^4^. For example, cancer can be classified into subtypes based on the pervasive differences in their gene expression patterns, and thus to provide a cancer patient with precise treatment^5, 6^.

Many clustering approaches have been proposed to analyze gene expression data, such as *k*-means^7^, hierarchical clustering^8^, local self-organizing maps^9^, local adaptive clustering^10^ and so on. Tavazoie *et al*.^7^ applied *k*-means to group gene expression data by assigning a sample to its nearest centroid, which is calculated by averaging all samples in that cluster. Eisen *et al*.^8^ applied average-link hierarchical clustering to cluster yeast gene expression data. Hierarchical clustering iteratively merges two closest clusters from singleton clusters, or partitions clusters into sub-ones by taking all samples as the single initial cluster. Average-link method uses the average distance between members of two clusters. These approaches have enabled researchers to explore the association between biological mechanisms and different physiological states, as well as to identify gene expression signatures. These traditional approaches, however, separately group gene expression data from genes dimension only. They can not discover the patterns that similar genes exhibit similar behaviors only over a subset of conditions (or samples), or relevant samples exhibit similar expression profiles over a subset of genes^11^. Patients of a cancer subtype may show similar expression profiles on a number of genes, instead of all^5, 6^.

Bi-clustering becomes an alternative to traditional clustering approaches for gene expression data analysis. Bi-clustering (or co-clustering), simultaneously groups genes and samples, it aims at discovering the patterns (or bi-clusters) that some genes exhibit similar expression values only on a subset of conditions^12, 13^. One can obtain a set of genes co-regulated under a set of conditions via bi-clustering. Bi-clustering shows great potentiality to find biological significance patterns^14^, which usually include: with constant values in the entire bi-cluster, with constant values in rows, with constant values in columns, with additive constant values and with multiplicative coherent values^15, 16^.

Many bi-clustering techniques have been applied to gene expression data analysis^17^. Cheng *et al*.^12^ pioneered a bi-clustering solution for grouping gene expression data, whose exact solution is known as a NP-hard problem. To combat with this problem, they used a greedy search to discover bi-clusters with low mean-squared residue score. Particularly, they iteratively removed or added genes and conditions from gene expression data matrix to find a bi-cluster, whose mean-squared residue score is below a certain threshold. However, this iterative solution can only produce one bi-cluster at a time, and it is hard to set a suitable threshold. Bergmann *et al*.^18^ proposed an iterative signature algorithm to iteratively search bi-clusters based on two pre-determined thresholds, one for matrix rows (representing genes) and the other one for matrix columns (representing samples). Obviously, the specification of these two thresholds affects the composition of bi-clusters. Therefore, similar as the solution proposed by Cheng *et al*.^12^, this signature algorithm is also heavily dependent on suitable setting of thresholds.

Researchers also move toward concurrently discovering multiple bi-clusters at a time. For instance, bi-clustering based on graph theory^19, 20^, information theory^21^, statistical method^22^, matrix factorization^23^. Sun *et al*.^24^ contributed a heuristic algorithm called Biforce, which transforms the data matrix into a weighted bipartite graph and judges the connection between nodes by a user-specified similarity threshold. Next, Biforce edits the bipartite graph by deleting or inserting edges to obtain bi-clusters. Kluger *et al*.^20^ proposed a spectral bi-clustering algorithm to simultaneously group genes and samples to find distinctive patterns from gene expression data. This algorithm is based on the observation that the structure of gene expression data can be found in the eigenvectors across genes or samples. It firstly computes several largest left and right singular vectors of the normalized gene expression data matrix, and then uses normalized cut^25^ or *k*-means on the matrix reconstructed by the left and right eigenvectors to obtain the row and column labels. Shan *et al*.^22^ proposed Bayesian co-clustering (BCC). BCC assumes that the genes (or samples) of gene expression data are generated by a finite mixture of underlying probability distributions, i.e., multivariate normal distribution. The entry of gene expression data matrix can be generated by the joint distributions of genes and conditions. One advantage of BCC is that it computes the probability of genes (or samples) belonging to several bi-clusters, instead of exclusively partitioning genes (or samples) into only one bi-cluster, but BCC suffers from a long runtime cost on large scale gene expression data. Dhillon *et al*.^21^ proposed an information-theoretic bi-clustering algorithm. Likewise BCC, this algorithm views the entry of gene expression data matrix as the estimation of joint probability of row-column distributions and optimizes these distributions by maximizing mutual information between entries of bi-clusters. But this approach is restricted to non-negative data matrix. Murali *et al*.^26^ proposed the concept of conserved gene expression motifs (xMOTIFs), each motif is defined as a subset of genes whose expressions are simultaneously conserved for a subset of samples. xMOTIFS aims to discover large conserved gene motifs that cover all the samples and classes in the data matrix. Hochreiter *et al*.^27^ proposed a factor analysis bi-clustering (FABIA) algorithm based on multiplicative model, which accounts for the linear dependency between gene expression profiles and samples. Lazzeroni *et al*.^28^ proposed a plaid model bi-clustering (Plaid), the entries of each bi-cluster are modelled by a general additive model and extracted by row and column indicator variables.

To explore bi-clusters with coherent trends, Cho *et al*.^29^ proposed a minimum sum-squared residue co-clustering (MSSRCC) solution to identify bi-clusters. MSSRCC iteratively obtains row and column clusters by a *k*-means like algorithm on row and column dimensions while monotonically decreasing the sum-squared residue. MSSRCC can discover multiple bi-clusters with coherent trends, or constant values. Gene expression data are always with a limit number of samples but with thousands of genes^4^. Distance between samples turns to be isometric as the number of genes (or gene dimension) increase^30^. MSSRCC firstly reduces the gene dimension by choosing genes with large deviation of expression levels among samples, and then applies bi-clustering on the pre-selected gene expression data to identify bi-clusters. However, this selection may lose the information hidden in the gene expression data, since the biological sense is not always straight^31^.

More recently, molecular interaction networks are also incorporated into bi-clustering to improve the performance of discovering cancer subtypes^32–35^. Knowing the subtype of a cancer patient can provide directional clues for precise treatment. Hofree *et al*.^36^ proposed a network-based stratification method to integrate somatic cancer with gene interaction networks. This approach initially groups cancer patients with mutations in similar network regions and then performs bi-clustering on the gene expression profiles using graph-regularized non-negative matrix factorization^37^. Liu *et al*.^38^ proposed a network-assisted bi-clustering (NCIS) to identify cancer subtypes via semi-non-negative matrix factorization^37^. NCIS assigns weights to genes as the importance indicator of genes in the clustering process. The weight of each gene refers to both the gene interaction network and gene expression profiles. However, NCIS can only discover bi-clusters with constant values.

The identified bi-clusters by a bi-clustering algorithm depend on the adopted objective function of that algorithm^17^. MSSRCC uses sum-squared residue as the objective function but it does not incorporate the gene interaction network. NCIS assigns weights to genes by referring to both the absolute deviation of genes expression profiles among samples and gene interaction network, but NCIS can only find bi-clusters with constant values, since its objective function is to minimize the distance between all entries of a bi-cluster and the centroid, defined by average of all entries in that bi-cluster.

To simultaneously discover multiple bi-clusters with constant or coherent values and to synergy bi-clustering with gene interaction network for cancer subtypes discovery, we introduce a novel method called Network aided Bi-Clustering (NetBC for short). NetBC firstly assigns weights to genes based on the structure of gene interaction network and the deviation of gene expression profiles. Next, it iteratively optimizes sum-squared residue to generate the row and column indicative matrices by matrix factorization. After that, NetBC takes advantage of the row and column indicative matrices to generate bi-clusters. To quantitatively and comparatively study the performance of NetBC, we test NetBC and other related comparing methods on several publicly available cancer gene expression datasets from The Cancer Genome Atlas (TCGA) project^39^. We use the clinical features of patients to evaluate the performance because the true subtypes of these samples belonging to are unknown. Experimental results show that NetBC can better group patients into subtypes than comparing methods. We further conduct experiments on cancer gene expression datasets with known subtypes to comparatively study the performance of NetBC. NetBC again demonstrates better results than these methods.

## Results and Discussion

To comparatively evaluate the performance of the proposed NetBC, we compared NetBC with NCIS^38^, MSSRCC^29^, BCC^22^, Cheng and Church (CC)^12^, BiMax^14^, Biforce^24^, xMOTIFs^26^, FABIA^27^, and Plaid^28^. Since NCIS, MSSRCC and BCC aim to extract non-overlapping bi-clusters with checkerboard structure, we compare NetBC with NCIS, MSSRCC and BCC on separating samples on two large scale cancer gene expression datasets from TCGA^34^ and several cancer gene expression datasets with known subtypes. CC, BiMax, Biforce, xMOTIFs, FABIA, and Plaid aim to extract arbitrarily positioned overlapping bi-clusters, we compare NetBC with CC, BiMax, Biforce, xMOTIFs, FABIA, and Plaid by relevance and recovery on synthetic datasets with implanted bi-clusters.

### Determining the number of Gene clusters (*k*) and sample clusters (*d*)

Determining the number of clusters is a challenge for most clustering methods. Here we adopt a widely used method to find the number of gene clusters *k* (or sample clusters *d*) that best fits the gene expression data matrix^38, 40^. If the number of gene clusters *k* (or sample clusters *d*) is suitable, we would expect that the gene separation (or sample separation) would change very little in different runs. For each run, we define an adjacency matrix of genes **M**_*g*_ with size *m*×*m* and an adjacency matrix of samples **M**_*s*_ with size *n*×*n*, ${{\bf{M}}}_{g}(i,j)=1$ when gene *i* and gene *j* belong to the same cluster and ${{\bf{M}}}_{g}(i,j)=0$, otherwise. Similarly, ${{\bf{M}}}_{s}(i,j)=1$ when sample *i* and sample *j* belong to the same cluster and ${{\bf{M}}}_{s}(i,j)=0$, otherwise. The consensus matrices ${\bar{{\bf{M}}}}_{g}$ and ${\bar{{\bf{M}}}}_{s}$ are the average of many base ${{\bf{M}}}_{g}$ and ${{\bf{M}}}_{s}$, which are obtained by repeatedly running the clustering method. The entry of ${{\bf{M}}}_{g}$ (${{\bf{M}}}_{s}$) is among 0 and 1. ${{\bf{M}}}_{g}$ reflects the similarity between genes and $1-{{\bf{M}}}_{g}$ denotes the distance between genes. If the selection of *k* is suitable, ${{\bf{M}}}_{g}$ is rather stable among multiple runs. In other words, ${\bar{{\bf{M}}}}_{g}(i,j)$ is close to 0 or 1. If the selection of *d* is suitable, ${{\rm{M}}}_{s}$ will not sharply fluctuate in different runs. We use the cophenetic correlation coefficient $\rho ({\bar{{\bf{M}}}}_{g})$ and $\rho ({\bar{{\bf{M}}}}_{s})$ to evaluate the stability of the consensus matrix. The cophenetic correlation coefficient $\rho ({\bar{{\bf{M}}}}_{g})$ is obtained by calculating Pearson correlation between the distance matrix $1-\bar{{\bf{M}}}$ and the distance matrix obtained by the linkage used in the reordering of $\bar{{\bf{M}}}$ ^38, 40^. To determine suitable *k* and *d*, we evaluate the stability of bi-clustering results over a range of combinations with *k* and *d*. We select the combination of *k* and *d* that produces the largest $\frac{\rho ({\bar{{\bf{M}}}}_{g})+\rho ({\bar{{\bf{M}}}}_{s})}{2}$.

### Results on TCGA cancer gene expression data

To comparatively evaluate the performance of the proposed NetBC, we compare NetBC with other related and representative bi-clustering methods on separating samples, including NCIS^38^, MSSRCC^29^, and BCC^22^ on two large scale cancer gene expression data from TCGA^34^. Since all the selected comparing aim to extract non-overlapping bi-clusters with checkerboard structure, we can use the dependence test between different clinical features and the discovered subtypes to evaluate their performance.

The lung cancer gene expression data contains 1298 patients (samples) with gene expression profiles of 20530 genes. The cancer subtypes of these samples are unknown. For comparison, we adopts the clinical features to study the performance of NetBC and these comparing methods. The clinical features are survival analysis, percent lymphocyte, eml4 alk translocation performed, pathologic stage, percent tumor cells stage, percent tumor nuclei. We choose relapse-free survival (RFS) for survival analysis. RFS means the length of time after primary treatment to a cancer patient that survives without any signs or symptoms of that cancer. RFS is one way to measure how well the treatment works. Percent lymphocyte means different percentages of infiltration of lymphocyte. Eml4 alk translocation clinical feature means whether Eml4 gene and alk gene are fused, the fusion of these two genes can lead to lung cancer. Pathologic stage represents different stages of the cancer pathologic. Percent tumor cell stage represents the percentages of tumor cells in the total cells. Percent tumor nuclei stage represents the percentages of tumor nuclei in a malignant neoplasm specimen. After removing samples with missing data of clinical features, 486 samples with 20530 genes are remained in the lung cancer dataset.

The breast cancer gene expression dataset contains 1241 patients with 17814 genes. Removing the samples that lack of clinical data, we finally retain 416 samples with 17372 genes. The clinical features to evaluate these bi-clustering methods contain AJCC Stage, Converted stage, Node coded, Tumor coded, Percent normal cells, and Percent tumor nuclei. We also make survival analysis for breast cancer, but no method has a *p*-value smaller than 0.05, one possible reason is the insufficient clinical data. The AJCC stage represents the different stages of the cancer based on the present lymph nodes. Converted stage represents different stages of the cancer. Node coded means different Node status of patients. Tumor coded means different types of tumor. Percent normal cells represents different percentages of normal cells in the malignant neoplasm specimen. Percent tumor nuclei represents different percentages of the tumor nuclei in the malignant neoplasm specimen.

The gene interaction network used for experiments are combined with networks collected from BioGRID^41^ (version: 3.4.138, access date: July 1, 2016), HPRD^42^ (version: 9, access date: February 15, 2017) and STRING^43^ (version: 10.0, access date: February 15, 2017). To fairly compare NetBC with NCIS, the collected gene interaction network for both NetBC and NCIS is directed. Since the TCGA cancer gene expression data is too large, we use *k*-means to initialize the indicative matrix **R** and **C** of NetBC, NCIS and MSSRCC. The number of iterations for these methods is set as 300. We set *k* = 7 and *d* = 6 for the TCGA lung cancer gene expression data and *k* = 11 and *d* = 6 for the TCGA breast cancer gene expression data based on the cophenetic correlation coefficient over a range of combinations of *k* and *d* (*k* from 1 to 12, and *d* from 4 to 6).

**Figure 1:**
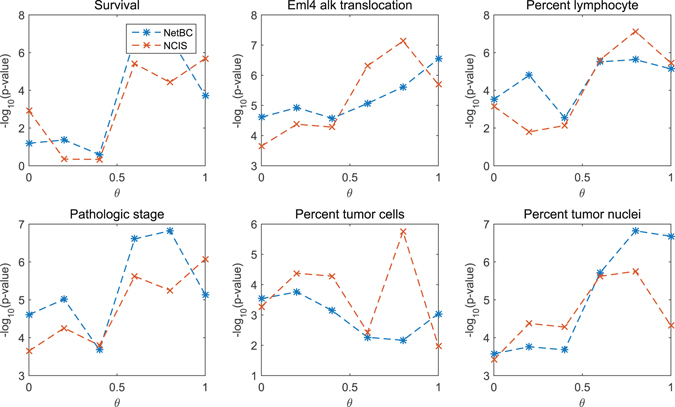
*p*-value of the dependence test between different clinical features and sub-types of Lung cancer discovered by NetBC (or NCIS) under different input values of *θ* For the survival time, we use logrank test. For the eml4 alk translocation performed, percent lymphocyte infiltration, percent tumor nuclei, and percent tumor nuclei, we use the Chi-squared test. The *p* value is adjusted by Benjamini & Hochberg method. Larger −log_10_(*p*-value) means better performance.

**Figure 2:**
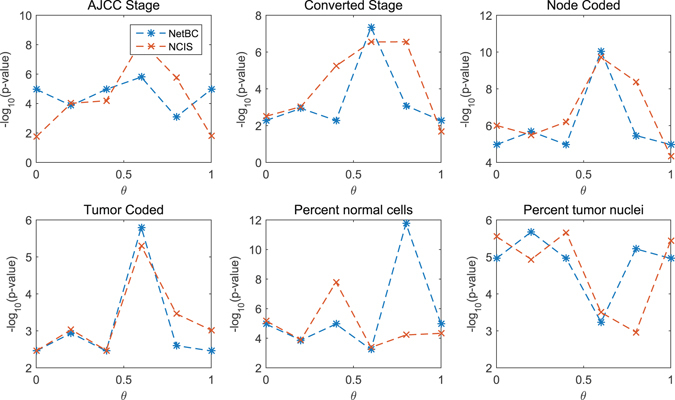
*p*-value of the dependence test between different clinical features and sub-types of Breast cancer discovered by NetBC (or NCIS) under different input values of *θ* For all the clinical features, we use the Chi-squared test. The *p* value is adjusted by Benjamini & Hochberg method. Larger −log_10_(*p*-value) means better performance.

*θ* is a scalar parameter to balance the contribution of gene interaction network and the deviation of gene expression profiles among samples when assigning genes weights. The significance levels of the difference between different clinical features and subtypes discovered by NetBC and NCIS with a range over different *θ* are given in Fig. 1 (Lung) and Fig. 2 (Breast). The *p*-value is adjusted by Benjamini & Hochberg method^44^. From Fig. 1 and Fig. 2, we can see that the input value of *θ* affects the experimental results of NetBC and NCIS. *θ* ∈ (0,1) means assigning weights to genes according to both the variation of gene expression levels and gene interaction network. We can find that NetBC and NCIS with *θ* ∈ (0.5, 1) show better performance than their cousins *θ* ∈ (0,0.5) in most cases. This observation demonstrates that assigning weights to genes according to both the variation of gene expression levels and gene interaction network can improve the performance of bi-clustering than using gene expression profiles along. NetBC also outperforms NCIS in majority cases.

To fairly compare NetBC, NCIS with BCC and MSSRCC, *θ* is setting as 0 for both NetBC and NCIS. Parameters of BCC are the number of gene clusters (or samples clusters), and the initialization of Gaussian distribution (*μ*, *σ*). *μ* and *σ* are fixed as random values provided in their demo codes, since there is no prior knowledge about the distribution of samples. The significance levels of the difference between subtypes discovered by these bi-clustering methods and the clinical features are given in Table 1 (Lung) and Table 2 (Breast). The *p*-value is adjusted by Benjamini & Hochberg method^44^. In these tables, a smaller *p* value indicates better results. From Table 1 and Table 2, we can see that NetBC has smaller *p*-value than other methods on most clinical features. Given the *p*-value threshold 0.05, NetBC successfully divides cancer patients into subtypes according to clinical features. BCC can not separate the cancer subtypes as well as others. That is principally because it assumes that patients are generated by a finite mixture of underlying probability distributions. Since the cancer gene expression data has limit samples with a large amount of genes, it is difficult to well estimate these underlying distributions. Although both NetBC and NCIS assign weights to genes as the importance indicator of genes, NetBC performs much better than NCIS on most clinical features. The main difference between them is that the objective function of NetBC is to minimize the sum-squared residue, while NCIS is to minimize the sum-squared distance between entries and centroids of bi-clusters. NCIS can only discover bi-cluster with constant values, NetBC can not only discover bi-clusters with constant values but also bi-clusters with coherent trend values. We can also observe that NetBC outperforms MSSRCC. MSSRCC utilizes a similar objective function as NetBC to minimize the sum-squared residue, the main difference between them is that NetBC assigns weights to genes, but MSSRCC does not. NetBC uses matrix factorization to get row and column indicative matrices and MSSRCC iteratively obtains row and column clusters by a *k*-means like algorithm on row and column dimensions. This observation shows that assigning weights to genes can improve the performance of bi-clustering.

**Table 1:** *p* value of the dependence test between different clinical features and the discovered subtypes of Lung cancer.

Method	Survival	Eml4 alk translocation	Percent lymphocyte	Pathologic stage	Percent tumor cells	Percent tumor nuclei
NetBC	3.03 × 10^−1^	**9.98 × 10** ^**−3**^	2.95 × 10^−2^	**9.98 × 10** ^**−3**^	**2.91 × 10** ^**−2**^	**2.79 × 10** ^**−2**^
NCIS	5.42 × 10^−2^	2.60 × 10^−2^	4.26 × 10^−2^	2.60 × 10^−2^	3.78 × 10^−2^	3.28 × 10^−2^
MSSRCC	**1.19 × 10** ^**−2**^	7.85 × 10^−2^	**2.48 × 10** ^**−2**^	4.34 × 10^−2^	6.30 × 10^−2^	5.23 × 10^−2^
BCC	8.54 × 10^−1^	3.78 × 10^−1^	8.54 × 10^−1^	8.54 × 10^−1^	9.97 × 10^−1^	7.57 × 10^−1^

For the survival time, we use logrank test. For the eml4 alk translocation performed, percent lymphocyte infiltration, percent tumor nuclei, and percent tumor nuclei, we use the Chi-squared test. The *p* value is adjusted by Benjamini & Hochberg method. The smaller the *p* value, the better the performance is.

**Table 2:** *p* value of the dependence test between different clinical features and the discovered subtypes of Breast cancer.

Method	AJCC Stage	Converted Stage	Node Coded	Tumor Coded	Percent normal cells	Percent tumor nuclei
NetBC	**6.96 × 10** ^**−3**^	1.02 × 10^−1^	**6.96 × 10** ^**−3**^	8.50 × 10^−2^	**7.01 × 10** ^**−3**^	**7.01 × 10** ^**−3**^
NCIS	1.80 × 10^−2^	4.82 × 10^−2^	4.11 × 10^−3^	**4.82 × 10** ^**−2**^	2.03 × 10^−2^	7.20 × 10^−3^
MSSRCC	2.30 × 10^−2^	**2.62 × 10** ^**−2**^	1.42 × 10^−2^	7.13 × 10^−2^	1.42 × 10^−2^	3.17 × 10^−2^
BCC	9.5 × 10^−1^	9.5 × 10^−1^	9.5 × 10^−1^	9.5 × 10^−1^	9.5 × 10^−1^	9.5 × 10^−1^

For all the clinical features, we use the Chi-squared test. The *p* value is adjusted by Benjamini & Hochberg method. The smaller the *p* value, the better the performance is.

### Results on cancer gene expression data with known subtypes

We also apply NetBC, NCIS, MSSRCC and BCC in clustering cancer gene expression datasets with known subtypes. Table 3 provides the brief description of these datasets. Breast contains three subtypes: samples from patients who developed distant metastases within 5 years (34 samples), samples from patients who continued to be disease-free after a period of at least 5 years (44 samples), samples from patients with BRCA germline mutation (20 samples). ALL contains three types of leukemia: 19 acute lymphoblastic leukemia (ALL) B-cell, 8 ALL (T-cell), 11 acute myeloid leukemia (AML). Liver contains four subtypes: sprague dawley (67 samples), wistar (32 samples), wistar kyoto (21 samples), fisher (2 samples). Leukemia contains three subtypes: ALL (B-cell) (10 samples), ALL (T-cell) (samples), AML (10 samples). Tumor contains two subtypes: cancer patients (31 samples) and normal (19 samples). DLBCLB includes three subtypes of diffuse large B cell lymphoma, ’oxidative phosphorylation’ (42 samples), ’B-cell response’ (51 samples), and ’host response’ (87 samples). DLBCLC contains four subtypes of diffuse large B cell lymphoma according to statistical differences of the survival analysis: 17 samples, 16 samples, 13 samples, 12 samples. MultiB contains four subtypes: breast cancer (5 samples), prostate cancer (9 samples), lung cancer (7 samples), and colon cancer (11 samples). The adopted gene interaction networks are also collected from BioGRID^41^, HPRD^42^ and STRING^43^.

**Table 3:** Details of 8 cancer gene expression datasets.

Dataset	Source	#Subtypes(d)	#samples(n)	#genes(m)
Breast	53	3	98	1213
ALL	54	3	38	5571
Leukemia	54	3	30	4412
Tumor	54	2	50	12422
Liver	55	4	122	8799
DLBCLB	56	3	180	661
DLBCLC	56	4	58	3759
MultiB	56	4	32	5565

#Subtypes is the number of cancer subtypes (or clusters), #samples is the number of samples, and #genes is the number of genes.

Since the ground truth sample clusters of these datasets are known, we adopt two widely used metrics: rand index (*RI*)^45^ and *F1-measure* ^46^ to evaluate the quality of clustering. Suppose the ground truth subtypes of samples in the gene expression data matrix are ${\mathscr{C}}=\{{{\mathscr{C}}}_{1},\mathrm{...},{{\mathscr{C}}}_{d}\}$, the clusters produced by a clustering method are ${\mathscr{C}}^{\prime} =\{{{\mathscr{C}}}_{1},\mathrm{...},{{\mathscr{C}}}_{{d}^{^{\prime} }}\}$. $n{p}_{1}$ represents the number of pairs of samples that are both in the same clusters of ${\mathscr{C}}$ and also both in the same clusters of ${\mathscr{C}}^{\prime}$; $n{p}_{2}$ represents the number of pairs of samples that are in the same clusters of ${\mathscr{C}}$ but in different clusters of ${\mathscr{C}}^{\prime}$; $n{p}_{3}$ represents the number of pairs of samples that are in different clusters of ${\mathscr{C}}$ but in the same clusters of ${\mathscr{C}}^{\prime}$; $n{p}_{4}$ represents the number of pairs of samples that are in different clusters of ${\mathscr{C}}$ and also in different clusters of ${\mathscr{C}}^{\prime}$. *RI* is defined as follows:

$$RI=\frac{n{p}_{1}+n{p}_{4}}{n{p}_{1}+n{p}_{2}+n{p}_{3}+n{p}_{4}}$$

*F1-measure* is the harmonic mean of precision and recall and is defined as follows:

$$F1-measure=\frac{2\,\ast \,Pr\,\ast \,Re}{Pr+Re}$$

where *Pr* and *Re* are precision and recall, defined as follows:

$$Pr=\frac{n{p}_{1}}{n{p}_{1}+n{p}_{3}}\quad \quad \quad Re=\frac{n{p}_{1}}{n{p}_{1}+n{p}_{2}}$$

**Figure 3:**
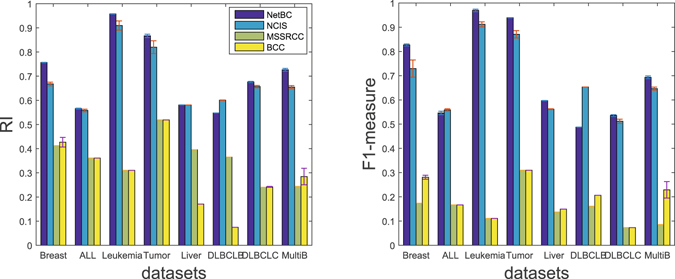
RI (**a**) and F1-measure (**b**) of different bi-clustering methods on eight datasets.

We compare the performance of NetBC with NCIS, MSSRCC and BCC. We perform the experiment by randomly initializing the row and column indicative matrices **R** and **C** of NetBC, NCIS and MSSRCC. The number of iterations for NetBC and NCIS is set as 300, and *θ* = 0 is used for both NetBC and NCIS. The parameter setting of BCC is similar to the experiment on TCGA cancer gene expression data. We perform 10 independent experiments and report the average results for each method on a particular dataset. We determine the number of gene clusters *k* based on the cophenetic correlation coefficient and set *d* as the ground truth number of cancer subtypes. Figure 3 provide the experimental results of these comparing methods with respect to RI and F1-measure. We can observe that NetBC performs better than other methods under both RI and F1-measure. This observation indicates that NetBC is an effective bi-clustering approach to identify cancer subtypes.

**Figure 4:**
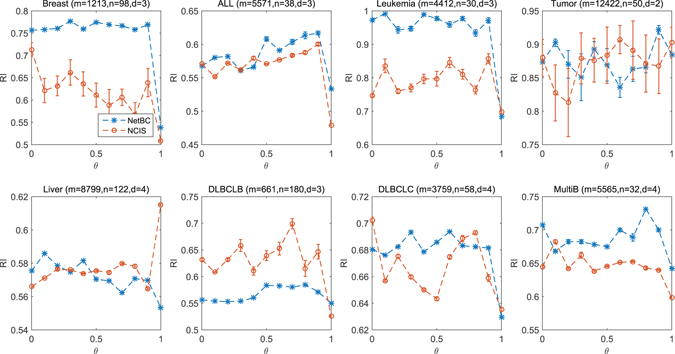
RI under different input values of *θ*.

**Figure 5:**
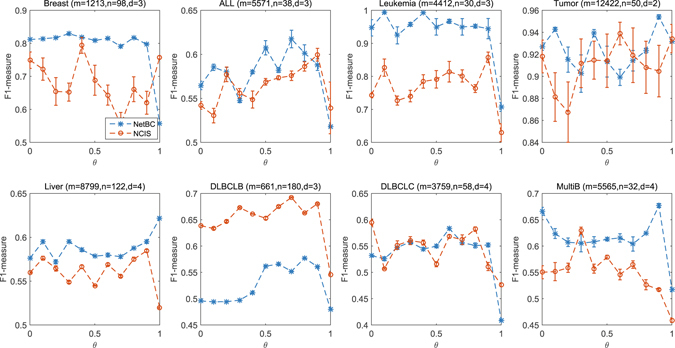
F1-measure under different input values of *θ*.

We also compare NetBC and NCIS when incorporating gene interaction network on real cancer gene expression data with known subtypes. *θ* controls the balance of referring to gene interaction network and the variation of gene expression profiles when assigning weights to genes. We analyze the influence of *θ* by varying its value from 0 to 1 with stepsize 0.1. We perform the experiment by randomly initializing the indicative matrices **R** and **C** of NetBC, NCIS, and fix the number of iterations for both NetBC and NCIS as 300. Figures 4 and 5 reveal the RI and F1-measure of NetBC and NCIS on eight cancer gene expression datasets. The reported experimental results are the average of ten independent runs for each particular dataset under each input value of *θ*. We can see that NetBC consistently outperforms NCIS over a range of *θ* values in most cases. This experimental results again demonstrate that NetBC improves the performance of cancer subtypes discovery. We can observe that NetBC (0.1 ≤ *θ* ≤ 0.9) generally has better performance than NetBC when *θ* = 0 (or *θ* = 1). From these observations, we can conclude that assigning weights to genes by referring to both gene expression profiles and gene interaction network shows advantage than assigning weights to genes by using gene interaction network (or by gene expression profiles) alone. However, there is no clear pattern to choose the most suitable *θ*. The possible reason is that *θ* is not only related to the deviation of expression profiles, but also the quality of gene interaction network and gene expression data. Adaptively choosing a suitable *θ* is an important future pursue. In summary, these empirical study shows that integrating gene interaction networks with gene expression profiles can generally boost the performance of bi-clustering in discovering cancer subtypes.

### Results on synthetic datasets

Let ${\mathcal E}$ denote the set of implanted bi-clusters and ${\mathcal E} ^{\prime}$ denote the set of bi-clusters discovered by a bi-clustering method. We can measure the similarity between the implanted bi-clusters and discovered bi-clusters by using the average bi-cluster relevance score^14^ as follows:

1 $${S}_{G}( {\mathcal E} ^{\prime} , {\mathcal E} )=\frac{1}{| {\mathcal E} ^{\prime} |}\sum _{({\mathscr{G}}^{\prime} ,{\mathscr{S}}^{\prime} )\in  {\mathcal E} ^{\prime} }\mathop{max}\limits_{({\mathscr{G}},{\mathscr{S}})\in  {\mathcal E} }\frac{{\mathscr{G}}^{\prime} \cap {\mathscr{G}}}{{\mathscr{G}}^{\prime} \cup {\mathscr{G}}}$$

where each ${\mathcal E} ^{\prime}$ (or ${\mathcal E}$) contains a gene set ${\mathscr{G}}^{\prime}$ (or ${\mathscr{G}}$) and a sample set ${\mathscr{S}}^{\prime}$ (or ${\mathscr{S}}$). The relevance score reflects to what extent the discovered bi-clusters represent implanted bi-clusters in the gene dimension. Similarly, average bi-cluster recovery is defined as ${S}_{G}( {\mathcal E} , {\mathcal E} ^{\prime} )$, which evaluates how well implanted bi-clusters are covered by a bi-clustering method.

Here, we compare NetBC with several bi-clustering methods on synthetic datasets with known bi-clusters. The codes of these comparing methods are online available, including Cheng and Church (CC)^12^, BiMax^14^, FABIA^27^, Plaid^28^, xMOTIFs^26^ and Biforce^24^.

Since NetBC and NCIS aim to discover non-overlapping bi-clusters, the synthetic datasets are generated without overlapping bi-clusters in the same way as that done by Prelic *et al*.^14^ and Wang *et al*.^47^ Particularly, these synthetic datasets are generated with five different types of bi-clusters, each synthetic dataset contains one type of bi-clusters. The five types of bi-clusters are (i) constant bi-cluster; (ii) row-constant bi-cluster; (iii) column-constant bi-cluster; (iv) additive coherent bi-cluster (or shift bi-cluster); (v) multiplicative coherent bi-cluster (or scale bi-cluster). The background matrices of these synthetic datasets are with entries randomly chosen from Gaussian distribution *N*(0, 1). Each bi-cluster is generated by choosing a submatrix from the background matrix with the entries modified according to one of the five rules: (i) constant bi-cluster is generated by randomly selecting an entry of a submatrix and replacing other entry values with this entry value; (ii) row-constant bi-cluster is generated by randomly selecting a base column within a selected submatrix and copying it to other columns in this submatrix; (iii) column-constant bi-cluster is generated by randomly selecting a base row within a selected submatrix and copying it to other rows in this submatrix; (iv) additive coherent bi-cluster is generated by randomly selecting a base row within a selected submatrix and replacing other rows in this submatrix by shifting the base rows; (v) multiplicative coherent bi-cluster is generated by randomly selecting a base row within a selected submatrix and replacing other rows in this submatrix by scaling the base rows. Then, we also add noise to these synthetic datasets to study the robustness of these comparing methods. Noise is simulated by adding random value from normal distribution to each entry of the synthetic gene expression data matrix. The noise level is increased by enlarging the standard deviation *σ* from 0.05 to 0.25 with stepsize 0.05.

It is crucial to select suitable parameters for bi-clustering tools. We follow the solution used by Eren *et al*.^48^ and Sun *et al*.^24, 49^ to select major parameters of these comparing bi-clustering tools over a range of values when they perform the best in the specified range. We set the number of the bi-clusters as the ground truth number of implanted bi-clusters for all bi-clustering approaches. *δ* and *α* are critical to the accuracy and runtime of CC. *δ* controls the maximum mean squared-residue in a bi-cluster. By default *δ* = 1, we run CC with different *δ* between 0 and 1 with stepsize 0.01. We set *δ* = 0.03, since CC performs the best when *δ* = 0.03. *α* controls the tradeoff between running time and accuracy. By default *α*=1, a larger *α* produces higher accuracy but asks for more runtime, we set *α* = 1.5 since the synthetic datasets are not too large. The number of max iterations for each layer in Plaid affects its results and its default value is 20, and we set it as 50 since Plaid performs best with the number of max iterations fixed as 50. We also select the row.release and column.release of Plaid in [0.5, 1] and set them as 0.6. BiMax and xMOTIFs are dependent on how the data are discretized. We performs xMOTIFs on synthetic data discretized with number of levels from 0 and 50 with stepsize 1. xMOTIFs performs best on synthetic data with the number of levels as 5. BiMax requires binary input data, we perform BiMax on synthetic data that are binary discretized with different thresholds from 0.05 to 1 with stepsize 0.05. BiMax performs best with threshold 0.1. We run Biforce with parameter *t*_0_ (edge threshold) from 0.05 to 1 with setpsize 0.05. Biforce performs best with *t*_0_ = 0.1. NetBC and NCIS depend on the initialization of indicative matrices **R** and **C**, we randomly initialize them for both NetBC and NCIS for all experiments on synthetic datasets. The number of iterations for NetBC and NCIS is set as 300. *θ* is set as 0, since the gene interaction networks of these synthetic datasets are not available.

**Figure 6:**
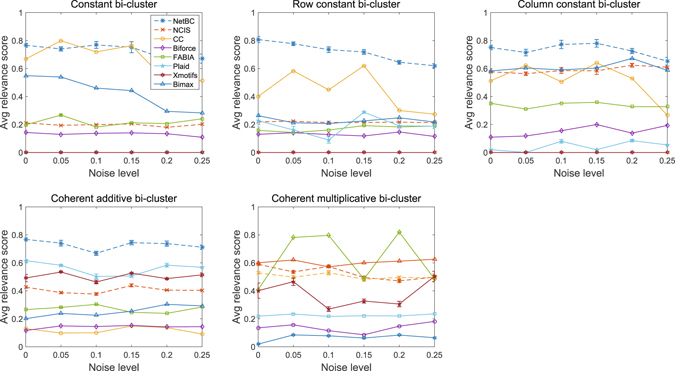
Relevance of bi-clustering methods on five types of bi-clusters.

**Figure 7:**
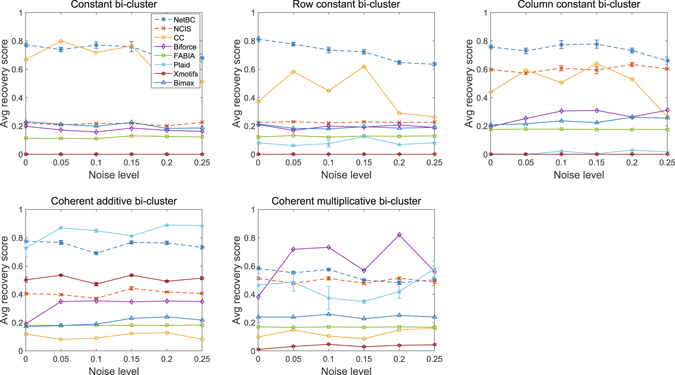
Recovery of bi-clustering methods on five types of bi-clusters.

From Figs 6 and 7, we can see that, even without incorporating gene interaction networks, NetBC still achieves better performance than most bi-clustering methods on the constant, row constant, column constant, coherent additive bi-clusters. CC achieves relatively better performance on constant, row constant and column constant bi-clusters. Plaid achieves better performance on coherent additive bi-clusters than other methods. xMOTIFs is skilled in extracting additive bi-clusters. Bimax is good at extracting column constant bi-clusters. On the coherent multiplicative bi-clusters, Biforce performs better than NetBC. Furthermore, we can see that NetBC outperforms NCIS on all these five types of bi-clusters and NetBC is generally robust to noise.

**Figure 8:**
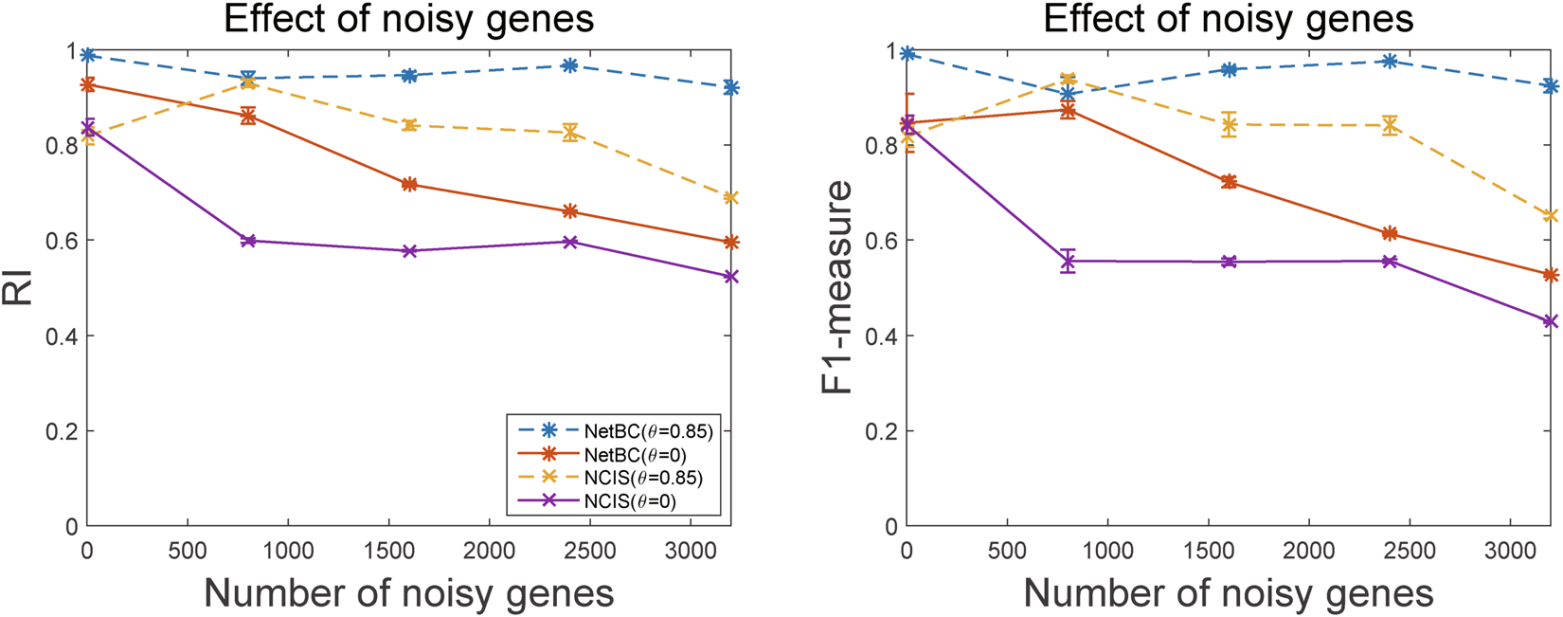
Performance of NetBC and NCIS under different number of noisy genes.

To further assess the performance of NetBC and NCIS, we compared their performance on separating samples in Leukemia cancer gene expression data with simulated noisy genes. Leukemia contains 38 samples and 4412 genes. The gene interaction network (obtained from BioGrid, HPRD and STRING) of Leukemia contains 162987 edges. We assign weights to genes according to the variation of gene expression profiles across samples and gene interaction network. Next, we choose genes with lowest weight as ‘uninformative’ genes and tag them as noisy genes. We permute the expression levels of these noisy genes with random numerics between the maximum and minimum value of the Leukemia gene expression data matrix. Figure 8 reports the results of NetBC and NCIS under different number of noisy genes.

From this Figure, we can see that NetBC almost always outperforms NCIS, but the performance of NetBC and NCIS with *θ* = 0, and of NCIS with *θ* = 0.85 downgrades as the number of noisy genes increase. The reason is that noisy genes not only mislead the variance of gene expression profiles, but also successively mislead the weights assigned to genes. We can find that NetBC and NCIS with *θ* = 0.85 generally have better performance than with *θ* = 0, and NetBC with *θ* = 0.85 is much less affected by noisy genes than NCIS with *θ* = 0.85. These experimental results demonstrate that incorporating gene interaction network to assign weight to genes can improve the performance of bi-clustering than assigning weights to genes based on the variation of gene expression profiles alone, and also show that NetBC can more effectively incorporating gene interaction network than NCIS.

**Figure 9:**
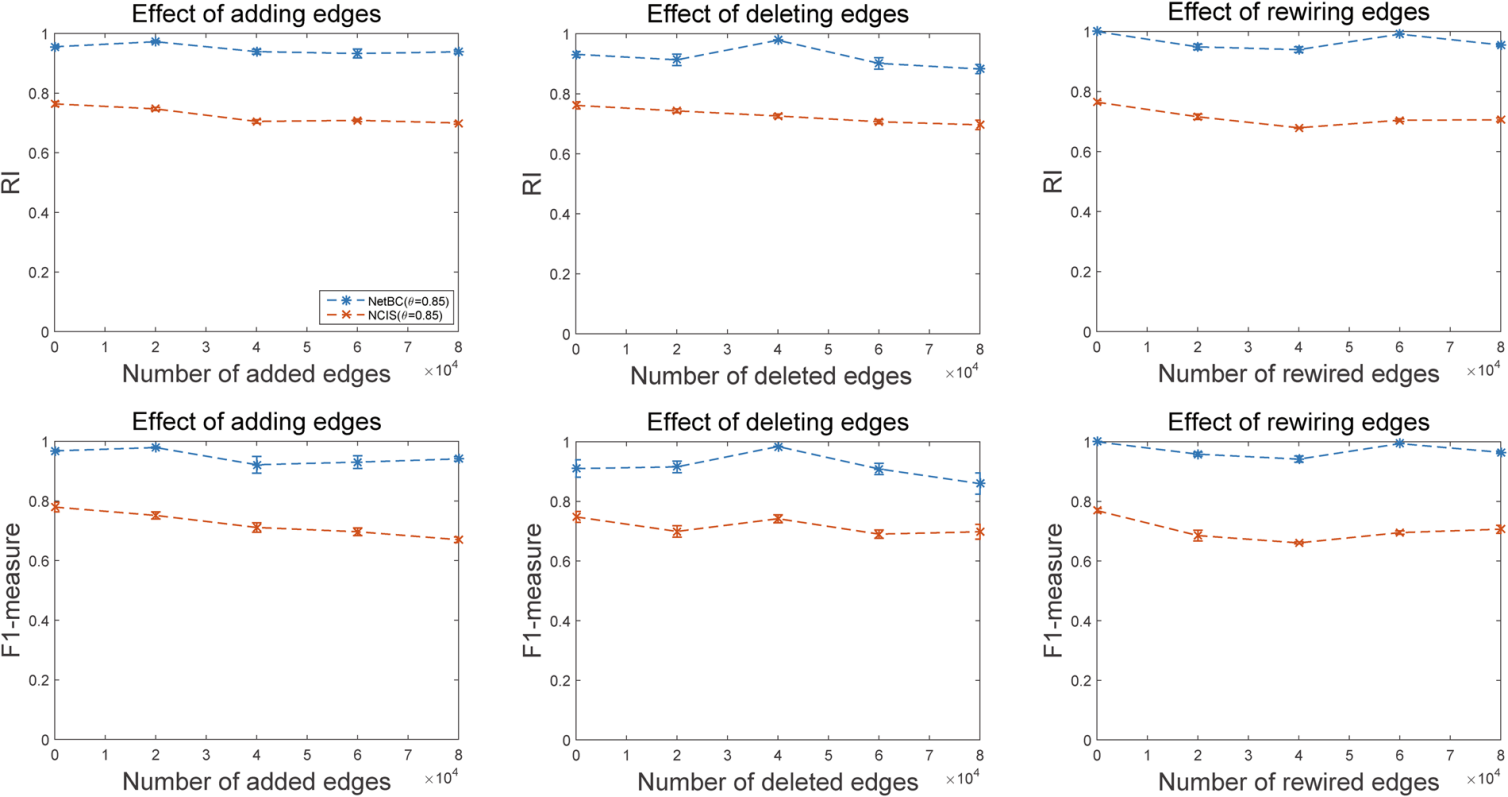
Performance of NetBC and NCIS with randomly added, deleted and rewired edges of gene interaction network.

We also explore the performance of NetBC and NCIS over random perturbations in the gene interaction network. Figure 9 reports the experimental results of NetBC and NCIS with *θ* = 0.85 on Leukemia dataset with randomly added, deleted and rewired edges between genes in the network. Here, NetBC and NCIS with *θ* = 0 are not considered, since *θ* = 0 means the network is excluded. By comparing the results in Fig. 8 with those in Fig. 9, we can see that, even with some fluctuations both NetBC and NCIS are relatively robust to perturbations of network. NetBC sometimes even obtains better results as more edges added or deleted. This observation is accountable, since the edges in the original network are not complete but with some noisy (missing) edges, and some randomly added (deleted or perturbed) edges just coincide with missing (or noisy) edges. We believe the performance of NetBC and NCIS can be further improved with the improved quality of gene interaction network.

### Runtime analysis

Bi-clustering is often involved with long runtime costs, especially when the gene expression data matrix is large. Therefore, it is highly challenging to develop an efficient bi-clustering algorithm. Suppose the number of iterations is *T*. In each iteration, NetBC takes $O({k}^{2}m+{k}^{3}+kmn)$ to compute ${\bf{R}}{\boldsymbol{(}}{{\bf{R}}}^{T}{\bf{R}}{{\boldsymbol{)}}}^{-1}{{\bf{R}}}^{T}{\bf{G}}$ (see Method Section for meanings of these matrix symbols), $O({n}^{2}d+kmn+{k}^{3})$ to compute ${\bf{W}}{{\bf{X}}}_{1}{{\bf{X}}}_{2}^{T}$, $O({k}^{2}m+kmn+k{m}^{2})$ to compute ${{\bf{X}}}_{3}^{T}{\bf{W}}{{\bf{X}}}_{4}$ and $O(dmn+{d}^{3}+d{n}^{2})$ to compute $\mathrm{GC}{\boldsymbol{(}}{{\bf{C}}}^{T}{\bf{C}}{{\boldsymbol{)}}}^{-1}{{\bf{C}}}^{T}$. Since $d\ll n$, $k\ll m$ and *m* is generally larger than *n*. The overall time complexity of NetBC is $O(T((k+d)mn+k{m}^{2}))$. NCIS also works on the same size matrices, it has similar time complexity as NetBC.

**Figure 10:**
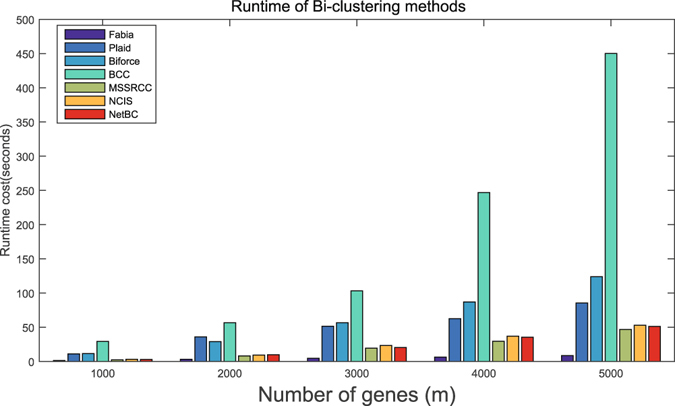
Runtime costs of eight bi-clustering methods under different number of genes, the number of samples is fixed as 200.

Here, we record the runtime change of NetBC and of several widely used bi-clustering methods as the number of genes increases from 1000 to 5000 and fixed the number samples as 200, and report the recorded results in Fig. 10. All the comparing methods are implemented on a desktop computer (Windows 7, 8GB RAM, Intel(R) Core(TM) i5-4590). The parameters of these methods are sets as default values. From Fig. 10, we can see that Fabia runs faster than other comparing methods. The runtime cost of NetBC is slightly larger than MSSRCC, since NetBC additionally utilizes gene interaction networks to assign weights to genes. NetBC is slightly faster than NCIS and they both increase relative slowly with the increase number of genes. The runtime cost of BCC is the largest and increases sharply, since BCC assumes the genes (or samples) of gene expression data are generated by a finite mixture of underlying probability distributions and it takes much time to estimate these distributions. Similarly, Plaid assumes the values of bi-clusters can be explained by additive model, it also needs relatively larger runtime cost than other comparing methods. The runtime cost of Biforce is larger than Plaid. In summary, NetBC can hold comparable efficiency with state-of-the-art bi-clustering methods and it generally obtains better performance than related methods.

## Methods

Let ${\bf{G}}\in {{\mathbb{R}}}^{m\times n}$ represent the gene expression data for *m* genes and *n* samples, the (*i*, *j*)-th entry of **G** is given by *g*_*ij*_. A bi-cluster is a subset of rows that exhibit similar behavior across a subset of columns, and it can be described as a sub-matrix of **G**. Let ${\mathcal I}$ represent a set of row indices for a row cluster and ${\mathscr{J}}$ represent a set of column indices for a column cluster. A sub-matrix ${{\bf{G}}}_{ {\mathcal I} {\mathscr{J}}}$ of **G** determined by ${\mathcal I}$ and ${\mathscr{J}}$ encodes a bi-cluster.

### Objective

The target of bi-clustering is to discover multiple bi-clusters from a gene expression data matrix, all the entries of a bi-cluster exhibit similar numerical values as much as possible. This target can be achieved by reordering the rows and columns of the matrix to group similar rows and similar columns together^13^. Traditional clustering algorithms group the similar genes (or samples) together by assigning genes (or samples) to their nearest cluster centroids. Ideally, entries are with constant value in the entire bi-cluster. For real world gene expression data, constant bi-clusters are usually distorted by noises. A common criterion to evaluate a bi-cluster is the sum of squared differences between each entry of a bi-cluster and the mean of that bi-cluster^17^. The squared difference between an entry *g*_*ij*_ and the mean of the corresponding bi-cluster is computed as below:

2 $${h}_{ij}={({g}_{ij}-{\tilde{g}}_{ {\mathcal I} {\mathscr{J}}})}^{2}$$

where ${\tilde{g}}_{ {\mathcal I} {\mathscr{J}}}=\frac{1}{| {\mathcal I} |}\frac{1}{|{\mathscr{J}}|}{\sum }_{i\in  {\mathcal I} ,j\in {\mathscr{J}}}{g}_{ij}$ is the mean of all entries in the bi-cluster, $| {\mathcal I} |$ is the cardinality of ${\mathcal I}$. Bi-clustering tries to find all combinations of ${\mathcal I}$ and ${\mathscr{J}}$, each combination has the minimum ${\sum }_{i\in  {\mathcal I} j\in {\mathscr{J}}}{h}_{ij}$.

**Figure 11:**
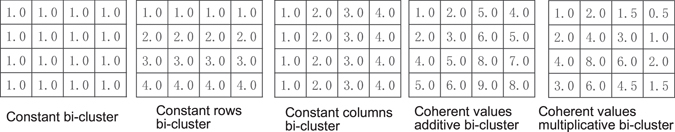
Example of the five types of bi-clusters.

In fact, the criterion to evaluate a bi-clustering method depends on the types of bi-clusters that can be identified by that method^15^. The criterion defined in Eq. (2) aims at finding bi-clusters with constant values. However, researchers may be not only interested with bi-clusters with constant values, but also bi-clusters with clear trends (or patterns), which generally include five major patterns^17^: (i) with constant values in the entire bi-cluster; (ii) with constant values in rows; (iii) with constant values in columns; (iv) with additive coherent values; (v) with multiplicative coherent values. If ${g}_{ij}=u(i\in  {\mathcal I} ,j\in {\mathscr{J}}\,)$, then $( {\mathcal I} ,{\mathscr{J}}\,)$ corresponds to a bi-cluster with constant value. If ${g}_{ij}=u+{\alpha }_{i}$ (or ${g}_{ij}=u{\alpha }_{i}$) with $i\in  {\mathcal I} ,j\in {\mathscr{J}}$, then $( {\mathcal I} ,{\mathscr{J}}\,)$ corresponds to a bi-cluster with constant value in rows. Similarly, if ${g}_{ij}=u+{\beta }_{j}$ (or ${g}_{ij}=u{\beta }_{j}$), then $( {\mathcal I} ,{\mathscr{J}}\,)$ corresponds to a bi-cluster with constant value in columns. If ${g}_{ij}=u+{\alpha }_{i}+{\beta }_{j}$ (or ${g}_{ij}=u\times {\alpha }_{i}\times {\beta }_{j}$), then $( {\mathcal I} ,{\mathscr{J}}\,)$ corresponds to a bi-cluster with additive (or multiplicative) coherent values. These five patterns of bi-clusters are recognized to have biological significance. Figure 11 provides illustrative examples for these five patterns of bi-clusters.

To discover bi-clusters of these patterns, we use the squared-residue to quantify the difference between an entry *g*_*ij*_ of a bi-cluster $( {\mathcal I} ,{\mathscr{J}})$ and the row mean, column mean and bi-cluster mean of that bi-cluster. Here we adopt a formula suggested by Cheng *et al*.^12^ as follows:

3 $${h}_{ij}={({g}_{ij}-{\tilde{g}}_{i{\mathscr{J}}}-{\tilde{g}}_{ {\mathcal I} j}+{\tilde{g}}_{ {\mathcal I} {\mathscr{J}}})}^{2}$$

where ${\tilde{g}}_{i{\mathscr{J}}}=\frac{1}{|{\mathscr{J}}|}{\sum }_{j\in {\mathscr{J}}}\,{g}_{ij}$ is the row mean, ${\tilde{g}}_{ {\mathcal I} j}=\frac{1}{| {\mathcal I} |}{\sum }_{i\in  {\mathcal I} }\,{g}_{ij}$ is the column mean, ${\tilde{g}}_{ {\mathcal I} {\mathscr{J}}}=\frac{1}{| {\mathcal I} |}\frac{1}{|{\mathscr{J}}|}{\sum }_{i\in  {\mathcal I} ,j\in {\mathscr{J}}}\,{g}_{ij}$ is the bi-cluster mean. The smaller the squared residue *h*_*ij*_, the larger the coherence is.

Suppose **G** is partitioned into *k* row (gene) clusters and *d* column (sample) clusters. We use ${\bf{R}}\in {{\mathbb{R}}}^{m\times k}({\sum }_{{k}^{^{\prime} }=1}^{k}{{\bf{R}}}_{i,{k}^{^{\prime} }}=1)$ as the indicative matrix for gene clusters, if ${{\bf{R}}}_{i{k}^{^{\prime} }}=1$, gene *i* belongs to gene cluster $k^{\prime}$. Similarly, we use matrix ${\bf{C}}\in {{\mathbb{R}}}^{n\times d}({\sum }_{{d}^{^{\prime} }=1}^{d}{{\bf{C}}}_{j,{d}^{^{\prime} }}=1)$ as the indicative matrix for sample clusters, if ${{\bf{C}}}_{j{l}^{^{\prime} }}=1$, sample *j* belongs to sample cluster *d*′. **R** and **C** has the following form: $${\rm{R}}=\mathop{\overbrace{[\begin{array}{cccc}1 & 0 & \cdots  & 0\\ 1 & 0 & \cdots  & 0\\ \vdots  & \vdots  & \cdots  & \vdots \\ 0 & 1 & \cdots  & 0\\ 0 & 1 & \cdots  & 0\\ \vdots  & \vdots  & \cdots  & \vdots \\ 0 & 0 & \cdots  & 1\\ 0 & 0 & \cdots  & 1\end{array}]}}\limits^{{\rm{k}}}\begin{array}{c}\\ \\ \\ \\ \\ \\ \\ \\ \\ \\ \\ \\ \\ \\ \\ \\ \\ \\ \\ \end{array}\}{\rm{m}}$$ $${\rm{C}}=\mathop{\overbrace{[\begin{array}{cccc}1 & 0 & \cdots  & 0\\ 1 & 0 & \cdots  & 0\\ \vdots  & \vdots  & \cdots  & \vdots \\ 0 & 1 & \cdots  & 0\\ 0 & 1 & \cdots  & 0\\ \vdots  & \vdots  & \cdots  & \vdots \\ 0 & 0 & \cdots  & 1\\ 0 & 0 & \cdots  & 1\end{array}]}}\limits^{{\rm{d}}}\begin{array}{c}\\ \\ \\ \\ \\ \\ \\ \\ \\ \\ \\ \\ \\ \\ \\ \\ \\ \\ \\ \end{array}\}{\rm{n}}$$

Assume row-cluster $k^{\prime}$ $(1\le k^{\prime} \le k)$ has ${m}_{{k}^{^{\prime} }}$ rows, and ${m}_{1}+{m}_{2}+\cdots +{m}_{k}=m$. Since there are *k* row clusters, we use ${ {\mathcal I} }_{{k}^{^{\prime} }}$ to represent the row index set of $k^{\prime}$-th row cluster. Then, we can obtain:

4 $${({\bf{R}}{({{\bf{R}}}^{T}{\bf{R}})}^{-1}{{\bf{R}}}^{T}{\bf{G}})}_{ij}=\sum _{k^{\prime} =1}^{k}{{\bf{R}}}_{i{k}^{^{\prime} }}{({{\bf{R}}}^{T}{\bf{R}})}_{{k}^{^{\prime} }{k}^{^{\prime} }}^{-1}{({{\bf{R}}}^{T}{\bf{G}})}_{{k}^{^{\prime} }j}=\sum _{{k}^{^{\prime} }=1}^{k}{{\bf{R}}}_{i{k}^{^{\prime} }}\frac{1}{{m}_{{k}^{^{\prime} }}}{({{\bf{R}}}^{T}{\bf{G}})}_{{k}^{^{\prime} }j},={\tilde{g}}_{ {\mathcal I} j}$$

where $${m}_{{k}^{^{\prime} }}=|{ {\mathcal I} }_{{k}^{^{\prime} }}|$$ and $${({{\bf{R}}}^{T}{\bf{G}})}_{{k}^{^{\prime} }j}={\sum }_{{i}^{^{\prime} }\in { {\mathcal I} }_{{k}^{^{\prime} }}}{{\bf{G}}}_{{i}^{^{\prime} }j}$$.

Similarly, we can obtain ${\bf{G}}{\bf{C}}{({{\bf{C}}}^{T}{\bf{C}})}^{-1}{{\bf{C}}}^{T}={\tilde{g}}_{i{\mathscr{J}}}$, ${\bf{R}}{({{\bf{R}}}^{T}{\bf{R}})}^{-1}{{\bf{R}}}^{T}{\bf{GC}}{({{\bf{C}}}^{T}{\bf{C}})}^{-1}{{\bf{C}}}^{T}={\tilde{g}}_{ {\mathcal I} {\mathscr{J}}}$. To explore multiple bi-clusters, NetBC minimizes the sum-squared residue over all genes and samples. Based on the above analysis, we can measure the overall sum-squared residue of multiple bi-clusters discovered by NetBC using **G**, **R** and **C** as follow:

5 $$\begin{array}{c}{\rm{\Psi }}({\bf{R}},{\bf{C}})=||{\bf{G}}-{\bf{R}}{({{\bf{R}}}^{T}{\bf{R}})}^{-1}{{\bf{R}}}^{T}{\bf{G}}-{\bf{GC}}{({{\bf{C}}}^{T}{\bf{C}})}^{-1}{{\bf{C}}}^{T}+{\bf{R}}{({{\bf{R}}}^{T}{\bf{R}})}^{-1}{{\bf{R}}}^{T}{\bf{GC}}{({{\bf{C}}}^{T}{\bf{C}})}^{-1}{{\bf{C}}}^{T}{||}^{2}\\ s\mathrm{.}t\mathrm{.}\,{{\bf{R}}}_{i{k}^{^{\prime} }}\geqslant 0,{{\bf{C}}}_{j{f}^{^{\prime} }}\geqslant 0,\sum _{k^{\prime} =1}^{k}{{\bf{R}}}_{i,{k}^{^{\prime} }}=1,\sum _{d^{\prime} =1}^{d}{{\bf{C}}}_{j,{d}^{^{\prime} }}=1.\end{array}$$

where ${({\bf{R}}{({{\bf{R}}}^{T}{\bf{R}})}^{-1}{{\bf{R}}}^{T}{\bf{G}})}_{ij}$ is the row mean, ${(\mathrm{GC}{\boldsymbol{(}}{{\bf{C}}}^{T}{\bf{C}}{{\boldsymbol{)}}}^{-1}{{\bf{C}}}^{T})}_{ij}$ is the column mean, and ${({\bf{R}}{({{\bf{R}}}^{T}{\bf{R}})}^{-1}{{\bf{R}}}^{T}{\bf{RGC}}{({{\bf{C}}}^{T}{\bf{C}})}^{-1}{{\bf{C}}}^{T})}_{ij}$ is the bi-cluster mean of (*i*, *j*) entry of **G** in its corresponding bi-cluster, respectively.

The above objective function of NetBC can also be reformulated as:

6 $${\rm{\Psi }}({\bf{R}},{\bf{C}})={\parallel {\boldsymbol{(}}{\bf{I}}-{\bf{R}}{\boldsymbol{(}}{{\bf{R}}}^{T}{\bf{R}}{{\boldsymbol{)}}}^{-1}{{\bf{R}}}^{T}{\boldsymbol{)}}{\bf{G}}{\boldsymbol{(}}{\bf{I}}-{\bf{C}}{\boldsymbol{(}}{{\bf{C}}}^{T}{\bf{C}}{{\boldsymbol{)}}}^{-1}{{\bf{C}}}^{T}{\boldsymbol{)}}\parallel }^{2}$$

where ${\bf{I}}\in {{\mathbb{R}}}^{m\times m}$ is an identity matrix, since ${\boldsymbol{(}}{\bf{I}}-{\bf{R}}{({{\bf{R}}}^{T}{\bf{R}})}^{-1}{{\bf{R}}}^{T}{\boldsymbol{)}}{\bf{G}}={\bf{G}}-{\bf{R}}{({{\bf{R}}}^{T}{\bf{R}})}^{-1}{{\bf{R}}}^{T}{\bf{G}}$.

### Assigning weights to genes

Gene expression data usually has a large amount of genes but with a few samples. The similarity between samples turns to be isometric as the gene dimensionality increasing^30^. Usually, genes are selected based on the absolute deviation value of the gene expression profile among samples to reduce the gene dimension. Feature selection methods, like principle component analysis (PCA)^50^, are applied to select genes to reduce the gene dimension. But selecting a subset of genes may result in information loss on clustering gene expression data especially when the biological sense is usually not straight. For this reason, assigning weights to genes to indicate the importance of the gene is more reasonable^38^. NetBC assigns weights to genes by using both the gene expression profiles and gene interaction network. Genes, who regulate more genes in the gene interaction network and show larger expression variations across samples than other genes, are viewed more important to identify cancer subtypes, and will be given larger weights.

NetBC uses the GeneRank algorithm^51^ to assign weights to genes. Suppose interactions between *m* genes are encoded by ${\bf{P}}\in {{\mathbb{R}}}^{m\times m}$. If there is directed (or undirected) an interaction from gene *i* to gene *j*, ${{\bf{P}}}_{ij}=1$ (or ${{\bf{P}}}_{ij}={{\bf{P}}}_{ji}=1$); otherwise, ${{\bf{P}}}_{ij}=0$. The importance of a gene depends on the quantity and importance of its interacting partners, a gene that interacts with more genes and shows larger variation of expression profiles across samples should be assigned with a larger weight. Based on this assumption, the weight is set as follows:

7 $${{\bf{w}}}_{i}^{t}=(1-\theta ){{\bf{e}}}_{i}+\theta \sum _{j=1}^{m}\frac{{{\bf{P}}}_{ij}{{\bf{w}}}_{j}^{t-1}}{de{g}_{j}},1\leqslant i\leqslant m$$

where ${{\bf{w}}}_{i}^{t}$ denotes the weight of gene *i* in the *t*-th iteration, **e**_*i*_ is the absolute value of expression profiles change for gene *i* among all samples. $de{g}_{j}={\sum }_{i=1}^{m}{{\bf{P}}}_{ij}$ means the total number of genes that have interactions with gene *j*. *θ* balances the weight from gene expression profiles and gene interaction network, it also enables isolated genes to be accessed and it is usually set as 0.85. Eq. (7) is guaranteed to convergence when 0 ≤ *θ* ≤ 1^38^. The final optimized weights for all genes in Eq. (7) can be computed as follows:

8 $$({\bf{I}}-\theta {\bf{P}}{{\bf{D}}}^{-1}){\bf{w}}=(1-\theta ){\bf{e}}$$

where ${\bf{D}}\in {{\mathbb{R}}}^{m\times m}$ is a diagonal matrix with the *j*-th diagonal element equal to $de{g}_{j}$. When $0 < \theta  < 1$, the weights of the genes are assigned according to Eq. (8). When *θ* = 0, the weights of genes are completely dependent on the deviation of gene expression profiles. When *θ* = 1, the weights of genes are assigned only based on the interacting partners of genes in the interaction network.

### Optimization

After assigned weights to genes, the objective function of NetBC can be rewritten as:

9 $${\rm{\Phi }}({\bf{R}},{\bf{C}})={\parallel {\boldsymbol{(}}{\bf{I}}-{\bf{R}}{\boldsymbol{(}}{{\bf{R}}}^{T}{\bf{R}}{{\boldsymbol{)}}}^{-1}{{\bf{R}}}^{T}{\boldsymbol{)}}{\bf{G}}{\boldsymbol{(}}{\bf{I}}-{\bf{C}}{\boldsymbol{(}}{{\bf{C}}}^{T}{\bf{C}}{{\boldsymbol{)}}}^{-1}{{\bf{C}}}^{T}{\boldsymbol{)}}\parallel }^{2}{\bf{W}}$$

where ${\bf{W}}\in {{\mathbb{R}}}^{n\times n}$ is diagonal matrix with ${{\bf{W}}}_{ii}={{\bf{w}}}_{i}$.

To optimize Eq. (9), we can iteratively optimize row indicative matrix **R** and column indicative matrix **C** by alternatively fixing one of them as constant. Let ${{\bf{X}}}_{1}={\bf{G}}{\boldsymbol{(}}{\bf{I}}-{\bf{C}}{\boldsymbol{(}}{{\bf{C}}}^{T}{\bf{C}}{{\boldsymbol{)}}}^{-1}{{\bf{C}}}^{T}{\boldsymbol{)}}$, ${{\bf{X}}}_{2}={({{\bf{R}}}^{T}{\bf{R}})}^{-1}{{\bf{R}}}^{T}{{\bf{X}}}_{1}$, then the objective function to optimize **R** can be rewritten as follows:

10 $$\begin{array}{rcl}Q({\bf{R}}) & = & \sum _{i=1}^{m}{\Vert {({{\bf{X}}}_{1})}_{i\mathrm{.}}-{({\bf{R}}{{\bf{X}}}_{2})}_{i\mathrm{.}}\Vert }^{2}{{\bf{W}}}_{ii}\\  & = & tr({{\bf{X}}}_{1}^{T}{\bf{W}}{{\bf{X}}}_{1}-2{{\bf{X}}}_{1}^{T}{\bf{WR}}{{\bf{X}}}_{2}+{{\bf{X}}}_{2}^{T}{{\bf{R}}}^{T}{\bf{WR}}{{\bf{X}}}_{2})\\  &  & s\mathrm{.}t\mathrm{.}\quad \quad {\bf{R}}\geqslant 0,\quad \sum _{k^{\prime} =1}^{k}{{\bf{R}}}_{i,{k}^{^{\prime} }}=1.\end{array}$$

Let ${{\bf{L}}}_{1}\in {{\mathbb{R}}}^{m\times k}$ be the Lagrangian multipliers for ${\bf{R}}({\bf{R}}\geqslant 0)$, then the Lagrangian function for **R** is:

11 $$L({\bf{R}},{{\boldsymbol{\Lambda }}}_{1})=Q({\bf{R}})-tr({{\boldsymbol{\Lambda }}}_{1}{{\bf{R}}}^{T})$$

To solve **R**, we let $\frac{\partial L({\bf{R}})}{\partial {\bf{R}}}=0$. Based on Karush Kuhn-Tucker conditions^52^, we can get

12 $${(-{{\bf{A}}}^{+}+{{\bf{A}}}^{-}+{\bf{R}}{{\bf{B}}}^{+}-{\bf{R}}{{\bf{B}}}^{-})}_{ij}{{\bf{R}}}_{ij}=0$$

where ${\bf{A}}={\bf{W}}{{\bf{X}}}_{1}{{\bf{X}}}_{2}^{T}$, ${\bf{B}}={{\bf{X}}}_{2}{{\bf{X}}}_{2}^{T}$, ${{\bf{A}}}^{+}=\frac{|{\bf{A}}|+{\bf{A}}}{2}$ and ${{\bf{A}}}^{-}=\frac{|{\bf{A}}|-{\bf{A}}}{2}$, ${{\bf{B}}}^{+}$ and ${{\bf{B}}}^{-}$ are similarly defined as ${{\bf{A}}}^{+}$ and ${{\bf{A}}}^{-}$. Then we can obtain the optimal **R** as follows:

13 $${{\bf{R}}}_{i{k}^{^{\prime} }}={{\bf{R}}}_{ik^{\prime} }\sqrt{\frac{{({{\bf{A}}}^{+}+{\bf{WR}}{{\bf{B}}}^{-})}_{ik^{\prime} }}{{({{\bf{A}}}^{-}+{\bf{WR}}{{\bf{B}}}^{+})}_{ik^{\prime} }}}$$

After that we can fix **R** to update the column indicative matrix **C**. Similarly, we set ${{\bf{X}}}_{3}=({\bf{I}}-{\bf{R}}{\boldsymbol{(}}{{\bf{R}}}^{T}{\bf{R}}{{\boldsymbol{)}}}^{-1}{{\bf{R}}}^{T}){\bf{G}}$, ${{\bf{X}}}_{4}={{\bf{X}}}_{3}{\bf{C}}{({{\bf{C}}}^{T}{\bf{C}})}^{-1}$. Then the objective function to obtain optimal **C** is:

14 $$\begin{array}{rcl}Q({\bf{C}}) & = & \sum _{i=1}^{m}{\Vert {({{\bf{X}}}_{3})}_{i\mathrm{.}}-{({{\bf{X}}}_{4}{{\bf{C}}}^{T})}_{i\mathrm{.}}\Vert }^{2}{{\bf{W}}}_{ii}\\  & = & tr({{\bf{X}}}_{3}^{T}{\bf{W}}{{\bf{X}}}_{3}-2{{\bf{X}}}_{3}^{T}{\bf{W}}{{\bf{X}}}_{4}{{\bf{C}}}^{T}+{\bf{C}}{{\bf{X}}}_{4}^{T}{\bf{W}}{{\bf{X}}}_{4}{{\bf{C}}}^{T})\end{array}$$

Let ${{\boldsymbol{\Lambda }}}_{2}\in {{\mathbb{R}}}^{n\times d}$ be the Lagrangian multiplier for **C**, then the Lagrangian function for **C** is as below:

15 $$L({\bf{C}},{{\boldsymbol{\Lambda }}}_{2})=Q({\bf{C}})-tr({{\boldsymbol{\Lambda }}}_{2}{{\bf{C}}}^{T})$$

Similarly, we can obtain the optimal formula of **C**.

16 $${{\bf{C}}}_{j{d}^{^{\prime} }}={{\bf{C}}}_{j{d}^{^{\prime} }}\sqrt{\frac{{({{\bf{D}}}^{+}+{\bf{C}}{{\bf{F}}}^{-})}_{j{d}^{^{\prime} }}}{{({{\bf{D}}}^{-}+{\bf{C}}{{\bf{F}}}^{+})}_{j{d}^{^{\prime} }}}}$$

where ${\bf{D}}={{\bf{X}}}_{3}^{T}{\bf{W}}{{\bf{X}}}_{4}$, ${\bf{F}}={{\bf{X}}}_{4}^{T}{\bf{W}}{{\bf{X}}}_{4}$. The optimal **R** and **C** can be iteratively optimized via Eq. (13) and Eq. (16) until Φ(**R**, **C**) convergency.

### Data availability

The Matlab codes of NetBC can be accessed from http://mlda.swu.edu.cn/codes.php?name=NetBC.
